# San Antonio Breast Cancer Symposium 2007—Adjuvant endocrine therapy update: ATAC 100 highlights

**Published:** 2008-01

**Authors:** Anil A. Joy

**Affiliations:** Department of Oncology, University of Alberta, and Cross Cancer Institute, Edmonton, Alberta

The two main classes of adjuvant hormonal therapy used in the treatment of postmenopausal women with hormone receptor–positive breast cancer are selective estrogen receptor modulators (for example, tamoxifen) and the aromatase inhibitors (ais—for example, anastrozole, letrozole, exemestane). The “gold standard” of 5 years of adjuvant tamoxifen has clearly been challenged (and some would argue surpassed) by the accumulating evidence from the adjuvant ai trials. In addition to a tolerable side-effect profile, the ai treatment strategies (upfront, switch, or extended adjuvant) have all demonstrated improved disease-free survival over adjuvant tamoxifen. They also offer a reduced incidence of thromboembolic disease and endometrial pathology, but come at the price of increased musculoskeletal symptoms, osteopenia and osteoporosis, and an associated increased fracture rate.

Follow-up data from the Arimidex, Tamoxifen, Alone or in Combination (atac) trial were just presented at the 2007 San Antonio Breast Cancer Symposium (sabcs) and have recently been published^1^. With a median follow-up of 100 months, atac is the longest-running ai trial to date, and its data continue to demonstrate improved efficacy for 5 years of upfront anastrozole over tamoxifen alone. As was seen in the last atac update and in the tamoxifen overview data, the absolute difference in time to recurrence (ttr) continues to increase even after hormonal treatment is stopped at 5 years—the “carryover effect”:

- At 5 years: 2.8% ttr (anastrozole 9.7% vs. tamoxifen 12.5%)
- At 9 years: 4.8% ttr (anastrozole 17.0% vs. tamoxifen 21.8%)
- Recurrence rates remained lower on anastrozole as compared with tamoxifen after treatment completion [hazard ratio (hr): 0.75; 95% confidence interval: 0.61 to 0.94; *p* = 0.01]

Although a nonsignificant numeric excess of non-breast cancer deaths in patients without a breast cancer recurrence was noted in the anastrozole group, no difference in overall survival was observed (hr: 0.97; *p* = 0.7).

No unexpected toxicity data were reported, and after the intended duration of endocrine therapy had been completed, no “carryover” with respect to increased fracture incidence was seen. Patients actively receiving anastrozole over tamoxifen had a higher annual fracture rate (2.93% vs. 1.90% respectively; incidence rate ratio: 1.55; *p* < 0.0001), but no difference was observed in those rates after endocrine therapy was complete (1.56% vs. 1.51% respectively; incidence rate ratio: 1.03; *p* = 0.79). Bisphosphonate use overall—let alone prophylaxis—was low in the trial. The hope is that preemptive measures to identify and treat osteopenia and osteoporosis, as per emerging evidence and evolving clinical guidelines, can further reduce this specific risk, particularly during the active treatment phase.

Apart from a continued reduction in endometrial cancer incidence with anastrozole over tamoxifen (0.2% vs. 0.8% respectively), no statistically significant differences in secondary malignancy rates were observed. Also reassuring was the lack of observed differences in cardiovascular morbidity or mortality between the two groups.

Although the adjuvant ai trials have provided many answers thus far, many questions remain. No single “ideal” adjuvant endocrine strategy for any given individual—let alone a given population—has emerged. Given differences in trial design, patient populations, medications used, follow-up to date, and lack of a published direct ai strategy comparison, declaring a clear “winner” is difficult. Adding to the complexity, intriguing early follow-up data from the Adjuvant Tamoxifen Longer Against Shorter (atlas) trial presented at sabcs 2007 suggest that longer-term tamoxifen (10 years) may even challenge the original premise of “gold standard” 5 year tamoxifen therapy^2^.

Although trends are suggestive, no single ai trial or adjuvant ai hormonal treatment strategy has yet provided convincing data demonstrating a clear, large, improvement in overall survival beyond that achieved with 5 years of adjuvant tamoxifen alone.

All that being said, there is hope. Patients and their clinicians have never had more treatment options at their disposal. And although the therapeutic differences may appear modest, the Early Breast Cancer Trialists’ Collaborative Group (2005–2006) overview data^a^ and cancer mortality statistics 3,4 demonstrate that successive, modest individual improvements in early detection, local control, and systemic therapy together lead to considerably larger, clinically meaningful differences in efficacy and breast cancer survival, given time.

The hope is that, one day, clinicians can offer patients effective, targeted, minimally toxic, individualized therapy based on an integrated understanding of tumoural and patient genetics, pharmacogenetics, and resistance mechanisms, thereby maximizing the predictive and prognostic capabilities of the various technologies available. Until that day materializes, however, the oncology community can anticipate covering large distances with cumulative small steps. As long as we keep heading in the right direction, we are bound to reach our destination.

## Footnotes

a Peto R, for the Early Breast Cancer Trialists’ Collaborative Group. The worldwide overview: new results for systemic adjuvant therapies. Presentation at the 30th Annual San Antonia Breast Cancer Symposium; December 13–16, 2007; San Antonio, TX, U.S.A.
